# Starch/PCL composite nanofibers by co-axial electrospinning technique for biomedical applications

**DOI:** 10.1186/s12938-017-0334-y

**Published:** 2017-03-29

**Authors:** B. Komur, F. Bayrak, N. Ekren, M. S. Eroglu, F. N. Oktar, Z. A. Sinirlioglu, S. Yucel, O. Guler, O. Gunduz

**Affiliations:** 1 Kanuni Sultan Suleyman Training and Research Hospital, Turgut Ozal Street No.1, Halkalı, Kucukcekmece, 34303 Istanbul, Turkey; 2 Advanced Nanomaterials Research Laboratory, Department of Metallurgical and Materials Engineering, Marmara University, Goztepe Campus, 34722 Istanbul, Turkey; 3 Department of Metallurgical and Materials Engineering, Institute of Pure and Applied Sciences, Marmara University, Goztepe Campus, 34722 Istanbul, Turkey; 4 Department of Electrical and Electronics Engineering, Faculty of Technology, Marmara University, Goztepe Campus, 34722 Istanbul, Turkey; 5 Department of Chemical Engineering, Faculty of Engineering, Marmara University, Goztepe Campus, 34722 Istanbul, Turkey; 6 Department of Bioengineering, Faculty of Engineering, Marmara University, Goztepe Campus, 34722 Istanbul, Turkey; 7 Aysin Biotechnology Limited Company, Istanbul, Turkey; 8 Department of Bioengineering, Faculty of Chemical and Metallurgical Engineering, Yıldız Technical University, Davutpasa Campus, 34220 Istanbul, Turkey; 9 Department of Orthopedics and Traumatology, Faculty of Medicine, Istanbul Medipol University, Halic Campus, 34083 Istanbul, Turkey; 10 Department of Metallurgical and Materials Engineering, Faculty of Technology, Marmara University, Goztepe Campus, 34722 Istanbul, Turkey

**Keywords:** Nanofiber, Electrospinning, Starch, PCL, Wound dressing

## Abstract

**Background:**

In this study, starch and polycaprolactone (PCL), composite nanofibers were fabricated by co-axial needle electrospinning technique. Processing parameters such as polymer concentration, flow rate and voltage had a marked influence on the composite fiber diameter. Fourier transform infrared spectroscopy, scanning electron microscopy (SEM), mechanical and physical properties (such as density, viscosity and electrical conductivity) of the composite fibres were evaluated. Moreover, a cell culture test was performed in order to determine their cytotoxicity for wound dressing application.

**Results:**

The effect of starch ratio in the solution on the properties and morphological structure of the fibers produced was presented. With lower starch concentration values, the fibers have greater ultimate tensile strength characteristic (mostly 4 and 5 wt%). According to SEM results, it can be figured out that the nanofibers fabricated have good spinnability and morphology. The mean diameter of the fibers is about 150 nm. According to results of cell culture study, the finding can be determined that the increase of starch in the fiber also increases the cell viability.

**Conclusions:**

Composite nanofibers of starch/PCL have been prepared using a co-axial needle electrospinning technique. PCL was successfully encapsulated within starch. Fiber formation was observed for different ratio of starch. With several test, analysis and measurement performed, some important parameters such as quality and effectuality of each fiber obtained for wound dressing applications were discussed in detail.

## Background

Nanotechnology is a newly-emerging and up-to-date technology that has been classified as a vital scientific and commercial venture with economic benefits in global scale. By rapid development of technology in last decades, the knowledge on nanomaterial manufacturing techniques have been increased and this consequently promoted, research groups studying on several areas around the world, to focus more on the preparation or syntheses techniques of nanomaterials for a variety of applications. According to literature, it can easily be seen that various techniques are reported for the fabrication of nanomaterials for different purposes. These techniques include drawing-processing, template-assisted synthesis, self-assembly, solvent casting, phase separation, and electrospinning techniques [1–3]. Among the various techniques used and reported in the literature, in the scientific community as well as in industry, electrospinning has garnered significant attention because of its ability to fabricate nanostructures with unique and advantageous properties such as a high surface area and inter/intra fibrous porosity [4]. Another advantage of using electrospinning technique is that it is the only one that can produce at large scale continuous nanofibers for industrial applications [5, 6]. Since it has been used in the early twentieth (1900) century, several significant improvements have been made in the design of different instruments, miscellaneous materials and nanomaterials production [7].

Electrospun nonwoven provide high-surface area, micro/nano-porosity, and the ability to add drugs, polymers or biomolecules into the nanofibers [8]. There may be several alternative utilization types of electrospinning one of them is co-axial needle electrospinning. It can be evaluated as an innovative extension of electrospinning. Co-axial needle electrospinning depends on the fact that it enforces the formation of fibers with a core–shell structure. The principles, technique details and biomedical applications of coaxial-needle electrospinning have been reported in detail in literature [9, 10].

In this study, the preferred method is co-axial needle electrospinning and the aim is to use the final products, which are nanofibers, in biomedical applications such as wound dressing.

Open wounds have high tendency to infection and bacterial colonization. Therefore, the property of bacterial barrier is an important parameter that can specify the success of a wound covering material [11]. Barrier property of materials to micro-organism is a leading aspect in many biomedical applications like wound dressings [12]. A wide range of biomaterials has been utilized by medical practitioners to handle chronic wounds [13]. The traditional forms of wound dressings are non-resorbable gauze and/or sponge, which are made of nonwoven, cellulose [14]. The conventional wound dressings continued for over 40 years, which were then swapped by the advanced materials, which comprise of thin films that are permeable to vapor and gases [14–16].

According to research made in biomedical applications, it has been indicated that synthetic or natural agents can be successfully used in wound dressing applications. Both synthetic polymers and natural polymers include in their structure hydrolytically or enzymatically labile bonds or groups are degradable [17]. The advantages of synthetic polymers are obvious, including predictable properties, uniformity and can be tailored easily [18]. Despite of all these good properties, they are extremely expensive. Thus, it leads researcher to focus on natural polymers, which are inherently biodegradable [19] and can be promising candidates to meet different requirements.

Starch is the centre of attention among lots of natural polymers. It is obtained from carbon dioxide and water by photosynthesis in plants [20]. By means of its complete biodegradability [21], low cost and renewability [22], starch can be used in promising candidate for developing sustainable materials [23, 24]. Many efforts have been employed to develop starch-based polymers for conserving the petrochemical resources, reducing environmental impacts and searching more applications [25–27]. On the other hand, PCL poly (ε-caprolactone) or simply polycaprolactone is synthetic biodegradable aliphatic polyester which has attracted considerable attention in recent years, particularly in the biomedical applications [28]. A number of polymers have been worked for their compatibility to modify the thermal, rheological as well as biophysical properties of PCL, based on its application [29–36].

In this work, starch/PCL composite nanofibers were fabricated with the electrospinning technique. Several characterization tests such as density, viscosity, electrical conductivity were performed. In order to report the structure characterization and morphology analysis of nanofibers produced, Fourier transform infrared (FTIR) and scanning electron microscope (SEM) analysis were conducted, respectively. Furthermore, cell culture testing was carried out to be able to demonstrate that nanofibers fabricated have great potential for wound dressing application.

## Methods

### Materials

Starch derived from corn {(C_6_H_10_O_5_)_n_, average molecular weight Mw ~106 g mol^−1^, amylose:amylopectin 25–28:72–75%} and dimethyl foramide (DMF) were supplied by Sigma Aldrich, UK. Dimethyl sulfoxide (DMSO) which is colorless and clear liquid {(CH_3_)2SO}, average molar mass 78.1 and its storage conditions at between +15 °C and +30 °C) was supplied by Merck KGaA. The average molecular weight of PCL (Mw) is 80,000 g mol^−1^, which was purchased from Sigma-Aldrich and used with no extra treatment or purification.

### Preparation of polymer composite solutions

The polymer composite solution consists of starch and PCL. They were separately prepared by dissolving their characteristic solvents. Herein, starch with different concentration (4, 5, 6, 7, 8 and 10 wt%) was dissolved in DMSO by stirring and heating at the same time and cooled down to room temperature whereas PCL (10 wt%) was dissolved in DMF and the same procedure was performed for this solution. Since different solvents were used it is not possible to mix them and electrospin. Instead, different needle was utilized for each solution which is called as co-axial electrospinning process.

### Experimental setup

**Fig. 1:**
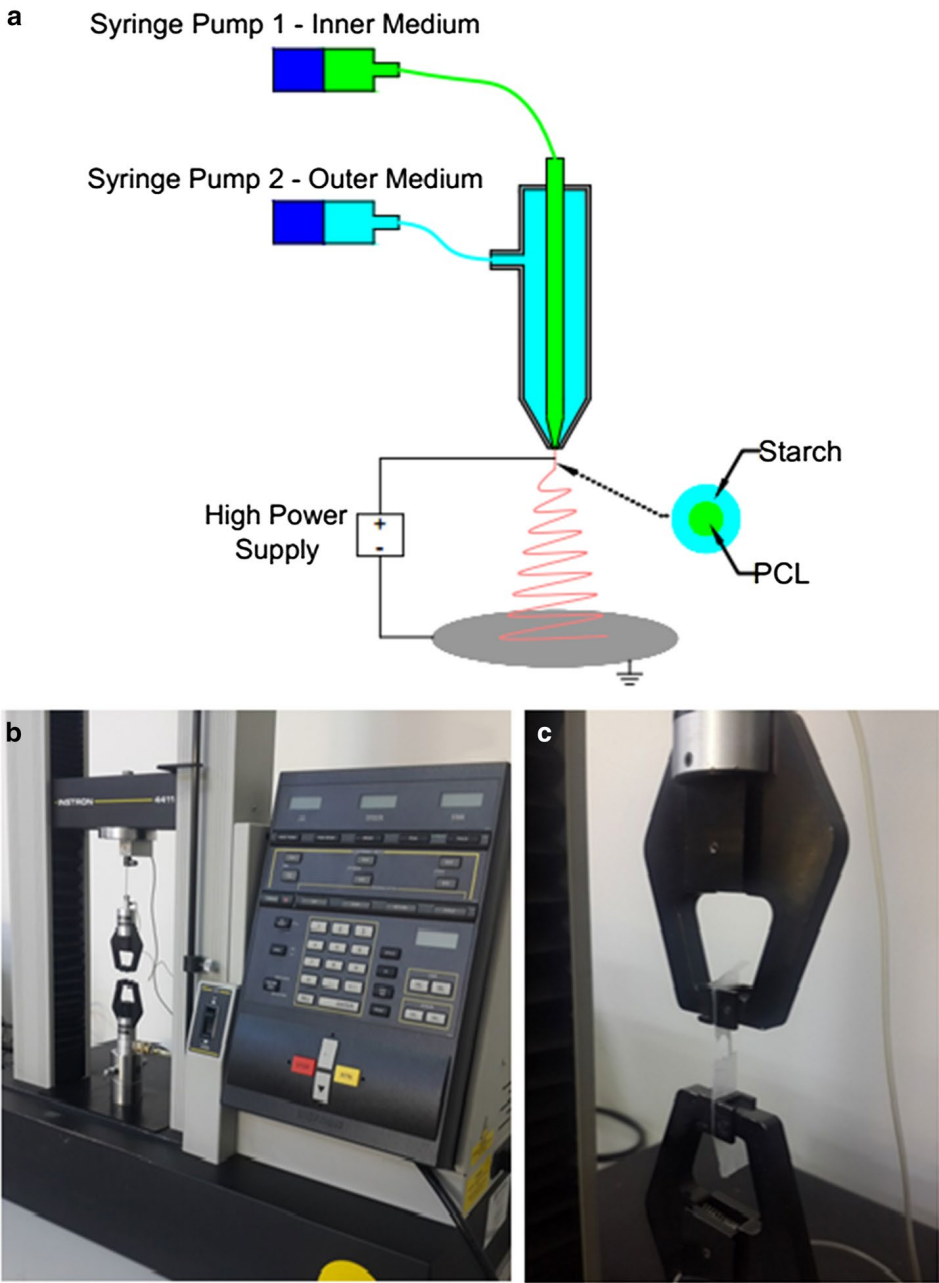
**a** Schematic representation of the co-axial needle electrospinning set-up. **b, c** Images of the Instron experimental set-up

The experimental set up used in this study is demonstrated in Fig. 1a. The co-axially arranged needles contained an outer needle with an internal diameter of 2700 µm and an external diameter of 3000 µm, and an inner needle with an internal diameter of 1560 µm and an external diameter of 2100 µm. Outer and inner stainless steel needles were connected to the same high-power voltage supply (Matsusada Precision, Shiga, Japan). The perfusion of PCL solution and starch solution into the individual needles was controlled by two syringe pumps (NE-300, New Era Pump Systems, Inc., New York, USA).

All experiments were performed at the ambient temperature. Processing a physically mixed composite solution was carried out by infusing the material into both the needles. The flow rates of these were fixed at a ratio of 1:2 for inner and outer needles, respectively. For encapsulation co-axial experiments, PCL solution was fed through the inner needle at half the flow rate of the starch solution. This solution was fed through the outer needle at various flow rates (15–25 µl min^−1^). At selected flow rates, from 25 kV up to 30 kV voltage was applied.

### Characterization

#### Rheology

In order to determine physical properties of solutions prepared, the values of density, viscosity and electrical conductivity were measured. Density was measured using a standard 10 ml density bottle. Viscosity was determined using a DV-E Viscometer (Brookfield AMETEK, Massachusetts, USA). Electrical conductivity was assessed using a conductivity meter whose model is Cond 3110 SET 1 (Xylem Analytics Germany Sales GmbH & Co. KG, WTW, Weilheim, Germany). All measurements were performed at the ambient temperature.

#### FT-IR analysis

FT-IR analysis was used to qualitatively characterize the functional groups of the electrospun fibers. The infrared (IR) spectra of starch/PCL composite samples were recorded on a Thermo Nicolet 6700 FTIR spectrophotometer equipped with Smart Orbit diamond ATR accessories. Measurements were conducted at room temperature between 400 and 4000 cm^−1^ in transmission mode.

#### Morphology

The morphological characterization of the starch/PCL composite nanofibers was examined using a scanning electron microscope (SEM; EVO LS 10, ZEISS). The pores, bead structures and nanofibers were observed and diameter range of fibers was determined on SmartSEM, which is the graphical user interface of the device. The samples were put on a metal stub using carbon adhesive tape and coated with gold under vacuum before observation.

#### Mechanical properties

Ultimate tensile strength (UTS) and elongation values were performed and measured by Instron 4411 tensile test machine by means of running a special software (Bluehill 2, Elancourt, France). The test speed of 5 mm min^−1^ and room temperature of (23 °C) were prepared. The cross-section is rectangular dimensions of 1 × 5 cm and the same for all test samples [37]. Three different samples were used to analyze each composite (with different percentages), therefore 18 different test samples were used during tensile tests. The distance between the grips was 1 cm to mount samples. Thickness of the composites was also measured (0035–0094 mm) using a digital micrometer (Mitutoyo MTI Corp). Photographs during mechanical tests are shown (Fig. 1b, c). The working area in which the study has been conducted was clean and experiments have been carried out at steady room temperature. Engineering diagrams have been obtained by a tensile test software and almost no deviation between the tests was observed.

#### Cell culture and cell viability

NIH-3T3 mouse fibroblast cell lines were used and cultured in DMEM with 10% FBS at 37 °C, 5% CO_2_. The culture medium was changed every second day. Cell viability was measured MTT assay according to manufacturer’s protocol (Sigma USA). Firstly all samples were sterilized with 70% ethanol and UV then small piece of membranes were laid down onto 96 well plate and cells were plated onto samples surface 103 cells per well in 96 well plate. Control was just tissue culture plate surface. After 1 and 3 days later 20 µl MTT solution (5 mg ml^−1^) was added into each well. After additional incubation for 4 h, 100 µl DMSO was added. The absorbance was read at 595 nm wavelength with microplate reader. Experiments were performed in triplicates. A test was performed to determine the statistical significance between experimental groups. Kruskal–Wallis test was used to compare the groups. A value of p < 0.05 was considered to be statistically significant.

## Results and discussions

The effect of starch concentration on the physical characteristics which are density, electrical conductivity and viscosity of starch solution was obtained and results are shown in the Table 1. It can be figured out that the higher the concentration of starch, the more electrical conductivity and viscosity. Similar observation is reported in literature for viscosity in which when such polymer concentration increases in the solution, the viscosity also increases proportionally [38]. This change can be easily observed. However, the addition of starch into the solution does affect the density lower than the other properties.

**Table 1:** Physical properties of solutions

Sample (w/w)	Density (g cm^−3^)	Viscosity (Pa s)	Electrical conductivity (µs cm^−1^)
4 wt% starch	1.106	0.35	7.00
5 wt% starch	1.113	0.72	8.20
6 wt% starch	1.115	0.98	8.56
7 wt% starch	1.118	1.28	9.80
8 wt% starch	1.119	1.45	10.00
10 wt% starch	1.126	4.65	11.26
10 wt% PCL	1.060	>500^a^	0.60

^a^Unit in cP

**Fig. 2:**
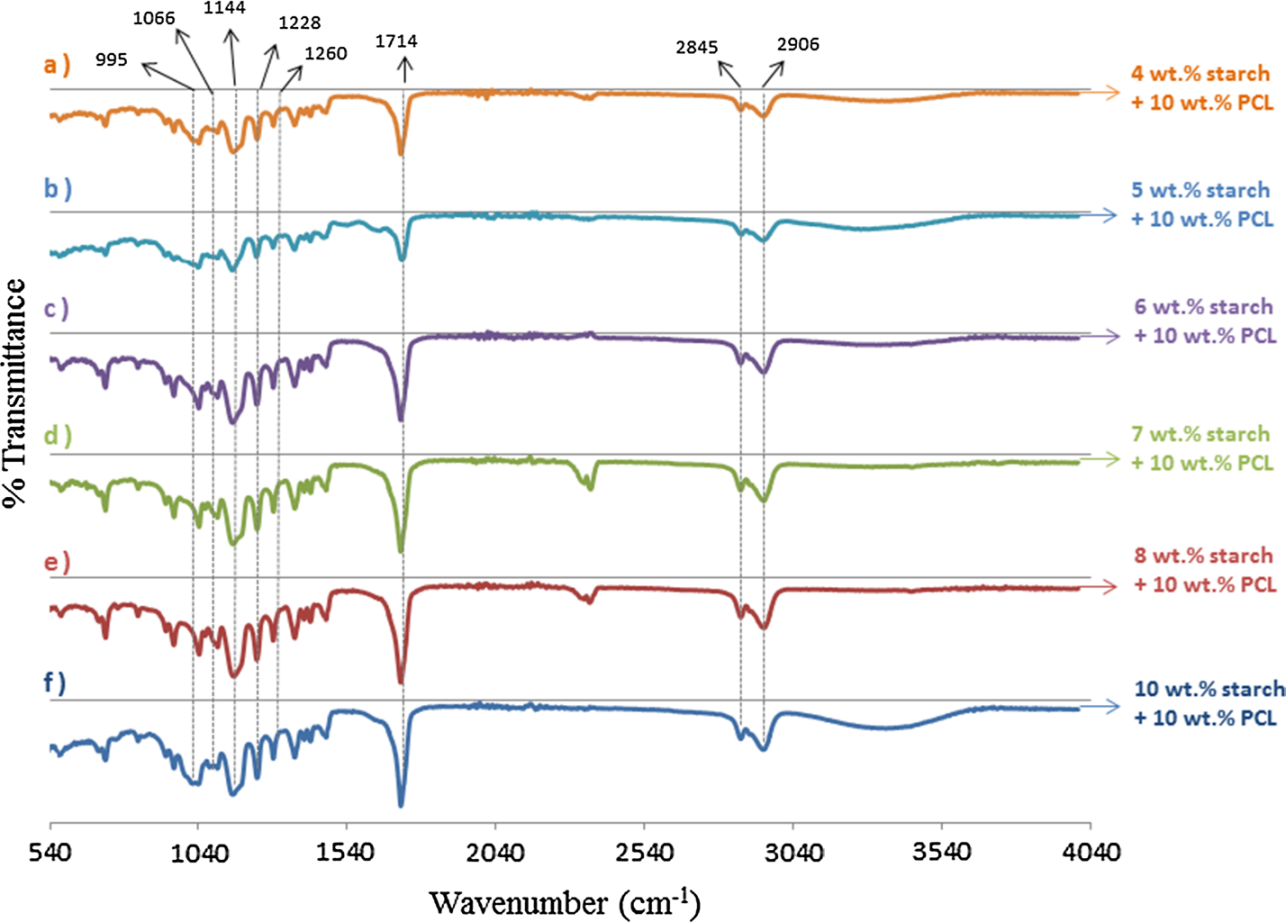
*a* 4 wt% starch/10 wt% PCL,* b* 5 wt% starch/10 wt% PCL,* c* 6 wt% starch/10 wt% PCL,* d* 7 wt% starch/10 wt% PCL,* e* 8 wt% starch/10 wt% PCL,* f *10 wt% starch/10 wt% PCL

Figure 2 reveals the wavenumber versus transmittance from (a) to (f) which correspond to the concentration of starch from 4 to 10 wt%, respectively. This figure represents the FTIR spectra of the starch/PCL composite nanofibers. It can be seen that there are three main peaks on the FT-IR spectra of the starch/PCL fibers on which the maximum absorbance is 1144, 1066 and 995 cm^−1^, respectively. While the bands at 1144 and 1066 cm^−1^ depend on the ordered structures of starch, the band at 995 cm^−1^ represents the starch that is amorphously structured [39]. The characteristic absorption bands at 1210 and 1720 cm^−1^ of starch were imputed to the bending vibration and stretching vibration of C–O, respectively. Moreover, the wide absorption band around 3423 cm^−1^ was because of the stretching vibration of O–H [40].

Several characteristic bands of PCL were observed at 2949 cm^−1^ (asymmetric CH2 stretching), 2865 cm^−1^ (symmetric CH2 stretching), 1727 cm^−1^ (carbonyl stretching), 1293 cm^−1^ (C\O and C\C stretching), 1240 cm^−1^ (asymmetric C\O\C stretching) and 1170 cm^−1^ (symmetric C\O\C stretching) [41].

**Fig. 3:**
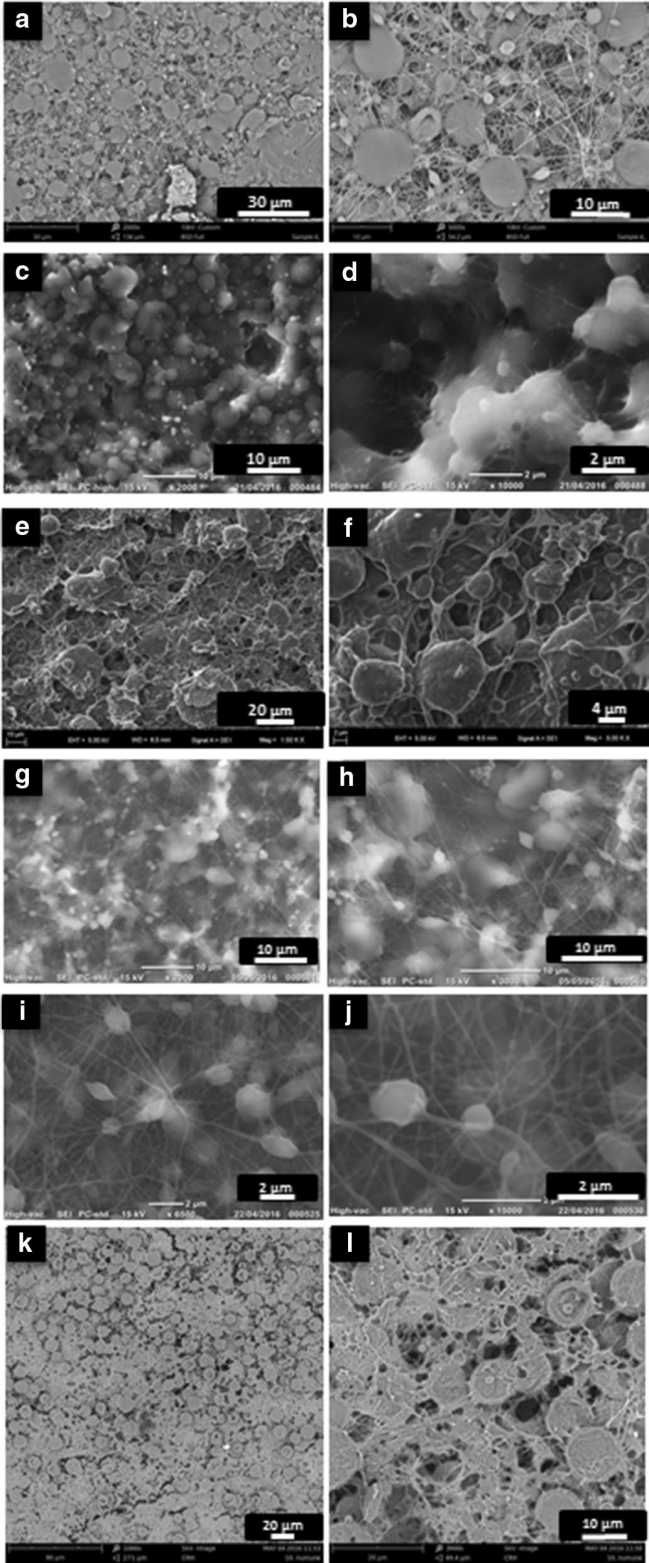
SEM images of the electrospun starch/PCL composite nanofibers with varying starch concentrations of **a**, **b** 4 wt% starch/10 wt% PCL composite fibers; **c**, **d** 5 wt% starch/10 wt% PCL; **e**, **f** 6 wt% starch/10 wt% PCL; **g**, **h** 7 wt% starch/10 wt% PCL; **i**, **j** 8 wt% starch/10 wt% PCL; **k**, **l** 10 wt% starch/10 wt% PCL

Morphological study has an importance to visualize and understand the orientation of structures inside the nanofibers fabricated. SEM image was used to observe the morphology of the starch/PCL composite nanofibers and it is shown in the Fig. 3 depending on the concentration of starch.

**Fig. 4:**
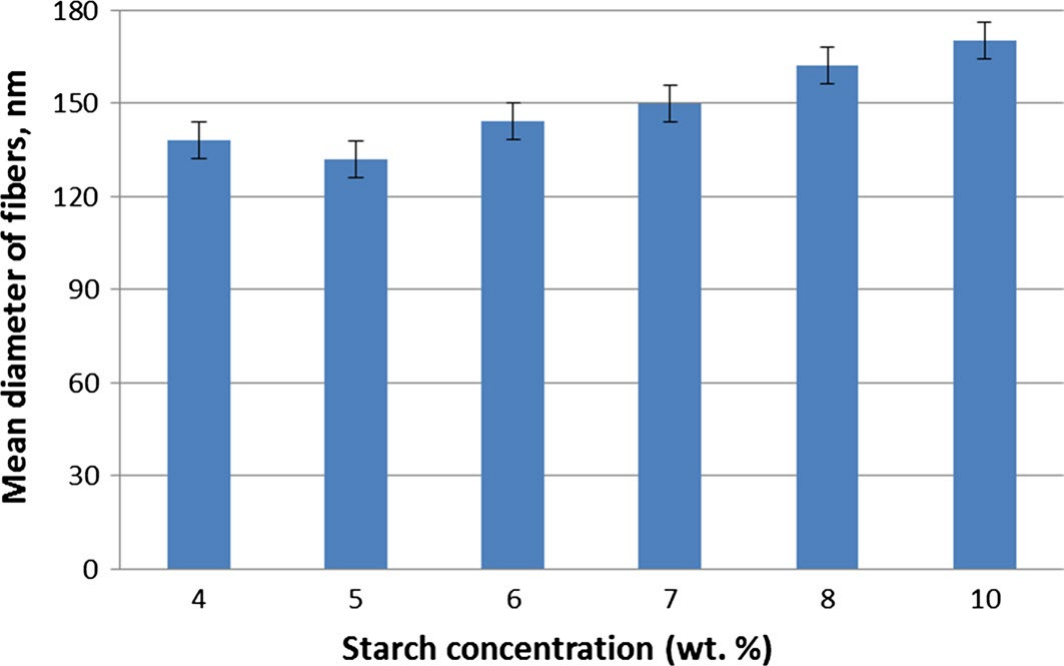
Mean diameter distribution of composite nanofibers with varying starch concentration mixed with 10 wt% PCL

In this Fig. 3, the subfigures that are associated with the low magnification results—Fig. 3a, c, e, g, i and k-show the general view of the nanocomposite structures and the fibers cannot be noticed very well. On the other hand, in others, high-magnification ones—Fig. 3b, d, f, h, j and l-, fibers and bead structures can be clearly observed. The conclusion that can be reached is that while PCL proportion in the electrospun nanocomposite structure is more prone to create fiber form, starch tends to turn into bead form. This is because of structural characteristics of starch. The bead diameter ranged from 958 to 1530 nm (Fig. 3a, b). The greatest bead diameter ranged from 2840 to 3530 nm (Fig. 3k, l). It was previously reported that some other material, such as PVA, is needed to be added to starch in order to increase its spinnability property [42]. Furthermore, the diameter range of nanofibers observed is 80–250 nm which is lower so better than the ones reported in the similar previous studies [43]. However, the general behavior of fiber diameter is to increase with the starch concentration (Fig. 4). This is because while the bead forms get bigger, the fibers connecting to these beads get thicker to provide a good strength. For instance, the fiber diameter is about 138 nm in the solution with 5 wt% starch/10 wt% PCL composite fibers (Fig. 3c, d) and it is about 150 nm in the solution with 7 wt% starch/10 wt% PCL composite fibers (Fig. 3g, h). As it can be seen, the mostly-measured diameter of fibers among entire the concentration values is about 150 nm (Fig. 4). A lower diameter range of fibers is caused by both low viscosity and low electrical conductivity. This is because the characteristic of low viscosity and electrical conductivity lead to a low viscoelastic force. [38]. In fiber diameter measurement performed in this study, results showed that the higher concentration of starch the higher viscosity (Table 1), fiber diameter and bead formation.

In order to perform tensile testing, the prepared nanofiber samples with different concentration of starch were used. While comparing the results to each other, the changing parameter was the concentration of starch which are 4, 5, 6, 7, 8 and 10 wt%, respectively. The mean value of Young’s modulus and strain at break were determined and listed in the Table 2.

**Table 2:** Young’s modulus and strain at break of each sample

PCL/starch nanofiber samples (wt%)	Young’s modulus (MPa)	Strain at break (%)
10 PCL/4 starch	10431.2	32.7
10 PCL/5 starch	10739.9	22.9
10 PCL/6 starch	7372.2	30.0
10 PCL/7 starch	6817.1	34.5
10 PCL/8 starch	6591.9	36.3
10 PCL/10 starch	4950.2	30.8

According to these results, it can be deduced that the Young’s modulus values are relatively higher for lower concentration values, such as 4 and 5 wt%, of starch in fibers (Table 2). Optimum starch concentration value may be determined as 5 wt% due to having the highest Young’s Modulus. It tends to decrease after this value because of the fact that the bead forms are bigger after the starch concentration of 5 wt% (Fig. 3c–l). The bigger form of beads, the lower value of Young’s Modulus. The values of strain at break associated with all concentration values are very close to one another. These characteristic behaviors are mostly based on the material properties so that different results have been observed in other similar studies performed with different materials and/or concentration in fibers [44].

**Fig. 5:**
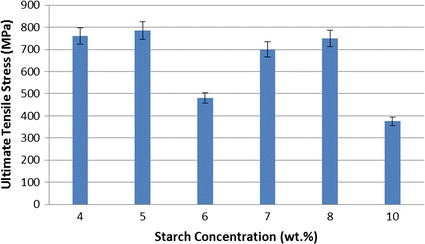
Ultimate tensile stress of composite nanofibers versus starch concentration

Ultimate tensile stress is another important factor to determine material characteristics of nanofibers produced. It represents the highest stress value at which nanofiber could hold without breaking. Ultimate tensile stress values for each sample are given in the Fig. 5.

Based on the analysis of ultimate tensile stress, it can be easily concluded that nanofibers’ ultimate tensile stress values somehow have a tendency to decrease with the increase of starch concentration. This shows that how the concentration of secondary materials affects the ultimate tensile stress of composite nanofibers produced. It depends on the properties of material added. Different type of materials and concentration values can give different results [45].

**Fig. 6:**
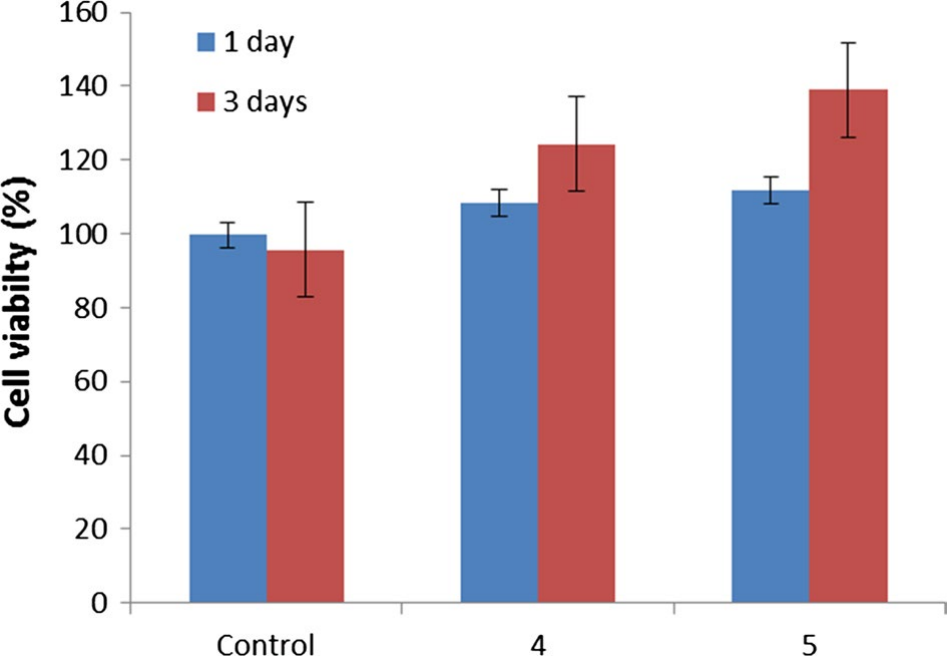
Cell viability of nanofiber with starch concentration of 4/10 wt% PCL and 5/10 wt% PCL

In order to carry out cell culture study, the composite nanofibers of 4 and 5 wt% starch were used. It was observed that the materials have no cytotoxic effect on fibroblast cells. The materials have increased the reproduction of cells since the first day. The material with the concentration of 5% increased the cell growth more than the one with the concentration of 4%. At the end of the third day, approximately, the material with the concentration of 4% increased the cell growth at the rate of 30% so did the one with concentration of 5% at the rate of 40%. The results are shown in Fig. 6.

According to results of cell culture study, the finding can be determined that the increase of starch in the fiber also increases the cell viability. However, the further analysis and longer time range are needed to demonstrate this finding more properly. To compare the result to the similar ones reported previously, addition of starch increases the cell growth more than pure PCL usage. Oh et al. [46] showed that PCL nanofibers increase the cell proliferation in order of approximately 20%. Depending on this, it can be concluded that greater increase of cell viability arises from saccharide increase in the medium produced from starch degradation [47].

## Conclusion

Composite nanofibers of starch/PCL have been prepared using a co-axial needle electrospinning technique. PCL was successfully encapsulated within starch. Fiber formation could have been observed for different ratio of starch. The effect of starch ratio in the solution on the properties and morphological structure of the fibers produced was presented. With lower starch concentration values, the fibers have greater ultimate tensile strength characteristic (mostly 4 and 5 wt%). According to SEM results, it can be figured out that the nanofibers fabricated have good spinnability and morphology. The mean diameter of the fibers is about 150 nm. According to results of cell culture study, the finding can be determined that the increase of starch in the fiber also increases the cell viability. However, such further analysis may be required to demonstrate this characteristic. Several characterization analysis have been performed and based on this, it was demonstrated that the starch/PCL composite nanofibers have great potential for wound dressing applications.

## Abbreviations

PCL: polycaprolactone
FTIR: Fourier transform infrared spectroscopy
SEM: scanning electron microscopy
DMF: dimethyl foramide
DMSO: dimethyl sulfoxide
UTS: ultimate tensile strength

## References

[1] Lim SK, Lee SK, Hwang SH, Kim H (2006). Photocatalytic deposition of silver nanoparticles onto organic/inorganic composite nanofibers. Macromol Mater Eng.

[2] Peng Y, Dong Y, Fan H, Chen P, Li Z, Jiang Q (2013). Preparation of polysulfone beads via vapor-induced phase separation and simulation of direct-contact bead distillation by measuring hydrophobic layer thickness. Desalination.

[3] Yongquan D, Ming W, Lin C, Mingjun L (2012). Preparation, characterization of P(VDF-HFP)/[bmim] BF4 ionic liquids hybrid beads and their pervaporation performance for ethyl acetate recovery from water. Desalination.

[4] Haider A (2015). A comprehensive review summarizing the effect of electrospinning parameters and potential applications of nanofibers in biomedical and biotechnology. Arab J Chem.

[5] Ramakrishna S, Fujihara K, Teo WE, Lim TC, Ma Z (2005). An introduction to electrospinning and nanofibers.

[6] Reneker DH, Yarin AL (2008). Electrospinning jets and polymer nanofibers. Polymer.

[7] Cooley JF. Improved methods of and apparatus or electrically separating the relatively volatile liquid component from the component of relatively fixed substances of composite fluids. United Kingdom Patent. 1900.

[8] Abrigo M, McArthur LS, Kingshott P (2014). Electrospun nanofibers as dressings for chronic wound care: advances, challenges and future prospects. Macromol Biosci.

[9] Moghe AK, Gupta BS (2008). Co-axial electrospinning for nanofiber structures: preparation and applications. Polym Rev.

[10] Amler E, Mickova A, Buzgo M (2013). Electrospun core/shell nanofibers: a promising system for cartilage and tissue engineering?. Nanomedicine.

[11] Augustine R, Kalarikkal N, Thomas S (2016). Electrospun PCL beads incorporated with biosynthesized silver nanoparticles as antibacterial wound dressings. Appl Nanosci.

[12] Augustine R, Kalarikkal N, Thomas S (2014). An in vitro method for the determination of microbial barrier property (MBP) of porous polymeric beads for skin substitute and wound dressing applications. Tissue Eng Regen Med.

[13] Augustine R, Kalarikkal N, Thomas S. Role of wound dressings in the management of chronic and acute diabetic wounds. In: George A, Augustine R, Sebastian M, editors. Diabetes mellitus and human health care: a holistic approach to diagnosis and treatment. Oakville: Apple Academic Press; 2014. pp 273–314.

[14] Augustine R, Kalarikkal N, Thomas S (2014). A facile and rapid method for the black pepper leaf mediated green synthesis of silver nanoparticles and the antimicrobial study. Appl Nanosci.

[15] Augustine R, Dominic EA, Reju I, Kaimal B, Kalarikkal N, Thomas S (2014). Investigation of angiogenesis and its mechanism using zinc oxide nanoparticle-loaded electrospun tissue engineering scaffolds. RSC Adv.

[16] Augustine R, Rajendran R, Cvelbar U, Mozetič M, George A. Biopolymers for health, food, and cosmetic applications. In: Thomas S, Durand D, Chassenieux C, Jyotishkumar P, editors. Handbook of biopolymer-based materials: from blends and composites to gels and complex networks. Weinheim: Wiley-VCH Verlag GmbH & Co. KGaA; 2013. pp 801–49.

[17] Lu DR, Xiao CM, Xu SJ (2009). Starch-based completely biodegradable polymer materials. Express Polym Lett.

[18] Nair LS, Laurencin CT (2007). Biodegradable polymers as biomaterials. Prog Polym Sci.

[19] Chiellini E, Solaro R (1996). Biodegradable polymeric materials. Adv Mater.

[20] Teramoto N, Motoyama T, Yosomiya R, Shibata M (2003). Synthesis, thermal properties, and biodegradability of propyl-etherified starch. Eur Polymer J.

[21] Araújo MA, Cunha A, Mota M (2004). Enzymatic degradation of starch-based thermoplastic compounds used in protheses: identification of the degradation products in solution. Biomaterials.

[22] Zhang JF, Sun XZ (2004). Mechanical properties of PLA/starch composites compatibilized by maleic anhydride. Biomacromol.

[23] Griffin GJL (1994). Starch polymer blends. Polym Degrad Stab.

[24] Pareta R, Edirisinghe MJ (2006). A novel method for the preparation of starch films and coatings. Carbohydr Polym.

[25] Park JS, Yang JH, Kim DH, Lee DH (2004). Degradability of expanded starch/PVA blends prepared using calcium carbonate as the expanding inhibitor. J Appl Polym Sci.

[26] Schwach E, Avérous L (2004). Starch-based biodegradable blends: morphology and interface properties. Polym Int.

[27] Stepto RFT (2006). Understanding the processing of thermoplastic starch. Macromol Symp.

[28] Azimi B, Nourpanah P, Rabiee M, Arbab S (2014). Poly (ε-caprolactone) fiber: an overview. J Eng Fibers Fabr.

[29] Liu CB, Gong CY, Huang MJ, Wang JW, Pan YF, Zhang YD, Li GZ, Gou ML, Tu MJ, Wang K, Wei YQ, Qian ZY (2008). Thermoreversible gel–sol behavior of biodegradable PCL-PEG-PCL triblock copolymer in aqueous solutions. J Biomed Mater Res B Appl Biomater.

[30] Kalambur S, Rizvi SSH (2006). Rheological behavior of starch–polycaprolactone (PCL) nanocomposite melts synthesized by reactive extrusion. Polym Eng Sci.

[31] Wan Y, Lu X, Dalai S, Zhang J (2009). Thermophysical properties of polycaprolactone/chitosan blend beads. Thermochim Acta.

[32] Ma Z, Haddadi A, Molavi O, Lavasanifar A, Lai R, Samuel J (2008). Micelles of poly(-ethylene oxide)-b-poly (epsilon– caprolactone) as vehicles for the solubilization, stabilization, and controlled delivery of curcumin. J Biomed Mater Res A.

[33] Sheikh FA, Barakat N, Kanjwal MA, Aryal S, Khil MS, Kim HY (2009). Novel selfassembled amphiphilic poly(epsilon–caprolactone)-grafted-poly(vinyl alcohol) nanoparticles: hydrophobic and hydrophilic drugs carrier nanoparticles. J Mater Sci Mater Med.

[34] Chen C, Cai G, Zhang H, Jiang H, Wang L (2010). Chitosan-poly(epsilon–caprolactone)- poly(ethylene glycol) graft copolymers: synthesis, self-assembly, and drug release behavior. J Biomed Mater Res A.

[35] Gorna K, Gogolewski S (2002). In vitro degradation of novel medical biodegradable aliphatic polyurethanes based on epsilon–caprolactone and pluronics with various hydrophilicities. Polym Degrad Stab.

[36] Pulkkinena M, Malin M, Böhmd J, Tarvainen T, Wirth T, Seppälä J, Järvinen K (2009). In vivo implantation of 2.2′-bis(oxazoline)- linked poly-epsilon–caprolactone: proof for enzyme sensitive surface erosion and biocompatibility. Eur J Pharm Sci.

[37] Croiser F, Duwez A-S, Jérôme C, Léonard AF, van der Werf KO, Dijkstra PJ, Bennink ML (2011). Mechanial testing of electropun PCL fibers. Acta Biomater.

[38] Shahreen L, Chase GG (2015). Effects of electrospinning solution properties on formation of beads in TiO_2_ fibers with PdO particles. J Eng Fibers Fabr..

[39] Sutjarittangtham K, Jaiturong P, Intatha U, Pengpat K, Eitssayeam S, Sirithunyalug J (2014). Fabrication of natural tapioca starch fibers by a modified electrospinning technique. Chiang Mai J Sci.

[40] Wang Q, Hu X, Du Y, Kennedy JF (2010). Alginate/starch blend fibers and their properties for drug controlled release. Carrbohyd Polym.

[41] Gautam S, Dinda AK, Mishra NC (2013). Fabrication and characterization of PCL/gelatin composite nanofibrous scaffold for tissue engineering applications by electrospinning method. Mater Sci Eng C-Mater.

[42] Liu Z (2014). Polyvinyl alcohol/starch composite nanofibers by bubble electrospinning. Therm Sci.

[43] Sutjarittangtham K, Jaiturong P, Intatha U, Pengpat K, Eitssayeam S, Sirithunyalug J (2014). Fabrication of natural tapioca starch fibers by a modified electrospinning technique. Chiang Mai J Sci.

[44] Croisiera F, Duwezb A-S, Jérômea C, Léonardc AF, van der Werfd KO, Dijkstrae PJ, Benninkd ML (2012). Mechanical testing of electrospun PCL fibers. Acta Biomater.

[45] Boakye MAD, Rijal NP, Adhikari U, Bhattarai N (2015). Fabrication and characterization of electrospun PCL-MgO-keratin-based composite nanofibers for biomedical applications. Materials..

[46] Oh GW, Ko SC, Je JY, Kim YM, Oh J, Jung WK (2016). Fabrication, characterization and determination of biological activities of poly(ε-caprolactone)/chitosan-caffeic acid composite fibrous mat for wound dressing application. Int J Biol Macromol.

[47] Mahdieh Z, Bagheri R, Eslami M, Amiri M, Shokrgozar MA, Mehrjoo M (2016). Thermoplastic starch/ethylene vinyl alcohol/forsterite nanocomposite as a candidate material for bone tissue engineering. Mater Sci Eng C.

